# Highly active antiretroviral therapy adherence among HIV-POSITIVE women in Southern Ethiopia

**DOI:** 10.3389/fphar.2024.1420979

**Published:** 2024-09-16

**Authors:** Alemayehu Abebe Demissie, Elsie Janse van Rensburg

**Affiliations:** ^1^ Department of Health Studies, University of South Africa (UNISA), Ethiopia Campus, Addis Ababa, Ethiopia; ^2^ Department of Public Health, College of Medicine and Health Sciences, Hawassa University, Hawassa, Ethiopia; ^3^ Department of Health Studies, University of South Africa (UNISA), Pretoria, South Africa

**Keywords:** adherence, barriers and facilitators, good adherence, highly active antiretroviral therapy, HIV-positive women

## Abstract

**Background:**

Adherence to Highly Active Antiretroviral Therapy (HAART) medication is the major predictor of HIV/AIDS treatment success. Poor adherence to HAART creates the risk of transmitting HIV, deteriorating health conditions, treatment failure, increased occurrences of drug-resistant HIV, morbidity and mortality. The objective of the study was to explore and describe factors influencing HAART adherence among HIV-positive women in Southern Ethiopia.

**Methods:**

A hospital-based descriptive cross-sectional survey was used among 220 randomly selected respondents. Data was collected with a structured interview guide after each respondent had given consent to take part in the study. The collected data was entered into and analyzed by using the Statistical Package for Social Sciences (SPSS) software program version 27.

**Results:**

The level of self-reported adherence (measured by dose) to HAART in the past 30 days was found to be 82.7%. In multivariate analysis, the divorced/separated HIV-positive women had poor adherence to their HAART medication as compared to those who were married [AOR: 2.94, 95% CI: (1.02–8.44)]. Respondents who used reminders in their medication were 75% less likely to be poorly adherent to their HAART medication than those who did not use reminders [AOR: 0.25, 95% CI: (0.06–0.97)]. Those who self-reported depression, perceived stigma, and low perceived susceptibility had poor adherence to their HAART than those who did not report depression, perceived stigma, and low perceived susceptibility [AOR:2.34, 95% CI: (1.01–5.42)], [AOR:2.37, 95% CI: (1.06–5.34)], and [AOR: 4.1, 95% CI: (1.53–11.1)] respectively. HIV-positive women who self-reported low perceived severity were poorly adherent to HAART than those who self-reported high perceived severity [AOR: 2.92, 95% CI: (1.14–7.47)].

**Conclusion:**

Factors including being divorced/separated, not using reminders, depression, perceived stigma, perceived susceptibility, and perceived severity negatively impact HIV-positive women’s adherence to HAART.

## 1 Introduction

The accessibility of HAART for people living with HIV/AIDS globally has increased rapidly in recent years to 28.7 million in 2021 from 7.8 million in 2010 (UNAIDS, 2022). Of all those ART-accessed people living with HIV/AIDS, only 68% of them were virally suppressed in 2021 (UNAIDS, 2022). As less viral suppression is associated with sub-optimal HAART adherence (DHHS, 2023; Zewude and Ajebe, 2022), the global less viral suppression (only 68%) in HIV-positive individuals indicates that sub-optimal (poor) adherence to HAART is a global challenge in HIV/AIDS treatment. Poor adherence to HAART medication has multiple negative effects on HIV-positive individual’s health conditions (Addo et al., 2022; Eribo and Adeleye, 2020). With the rapid scale-up of HAART worldwide, poor treatment adherence is a key challenge to antiretroviral therapy (ART) in sub-Saharan Africa (Angelo and Alemayehu, 2021). Sub-Saharan African countries are continuing to struggle with poor adherence to HIV/AIDS treatment (Azia, Mukumbang, Shernaaz & Nyembezi, 2022).

Ethiopia is one of the sub-Saharan African countries highly affected by HIV epidemics (Angelo and Alemayehu, 2021; Kibret, Ferede, Leshargie et al., 2019). HIV remains a major public health problem in Ethiopia (Angelo and Alemayehu, 2021). As per the Federal Ministry of Health (FMOH) report, the first confirmation of the HIV pandemic in Ethiopia was identified in 1984. Ever since acquired immune deficiency syndrome (AIDS) has killed millions of people and has left behind hundreds of thousands of orphans (FMOH, 2018). Due to this, the Ethiopian Government took numerous measures to avoid further disease spread and to expand the availability of HIV Care, treatment, and support for people living with HIV (FMOH, 2018). As per the report of the Ethiopian Demographic and Health Survey (EDHS), the national HIV prevalence is 0.9% and the female prevalence is 1.2% which is greater than that of the males which is 0.6% (CSA and ICF, 2016). Women covered 61.1% of all people who are HIV positive in Ethiopia and HIV is more prevalent, i.e., 1.62 times higher among adult women than men (Girum et al., 2018).

In Ethiopia, there were 414,854 patients, including 21,146 children under the age of 15, who were taking HAART in 2017 (FMOH, 2018). As to the 2017 projection, the national ART need was 551,630 for adults (FMOH, 2018). Highly active antiretroviral therapy (HAART) offered to HIV-positive individuals has tremendous life-sustaining benefits. These benefits are sustainable only through good HAART adherence (Nigusso and Mavhandu-Mudzusi, 2020). As mentioned by Nigusso and Mavhandu-Mudzusi (2020), poor adherence remains a major barrier to achieving the clinical and public health benefits of antiretroviral drugs (ARVs). In the Benshangule region of Ethiopia, a study in one urban public hospital that was conducted on men and women who are HIV positive revealed that from a total number of 259 women, about 110 (42.5%) of them were non-adhering to their medication (Nigusso and Mavhandu-Mudzusi, 2020).

Good adherence to HAART is necessary to achieve the best virological response, lower the risk that drug resistance will develop, and reduce morbidity and mortality (Angelo and Alemayehu, 2021; Nigusso and Mavhandu-Mudzusi, 2020). With the increased availability of HAART in recent years, the achievement of good adherence and patient retention are becoming the greatest challenges in the management of HIV/AIDS in Ethiopia (Angelo and Alemayehu, 2021).

A research study conducted in Ethiopia on 339 people diagnosed with HIV revealed that 25.4% had poor adherence to their medication (Gebreagziabher and Woldemariam, 2020). Similarly, another study done in Ethiopia showed that only 73.1% of patients were adherent to their HAART (Abadiga et al., 2020). Numerous factors have been mentioned as reasons for poor adherence in studies of sub-Saharan African countries (Lahai et al., 2022). The barriers and challenges to HAART adherence include factors associated with patients and their families, socioeconomic factors, medication, and health providers and systems (Lahai et al., 2022; Moomba and Van Wyk, 2019).

Patient adherence to ARV combination therapy is a critical component of successful treatment outcomes. Studies indicate that women have poor adherence as compared to men (Audrey et al., 2020). Women who are HIV positive face many challenges in ensuring adherence to HAART more than their male counterparts. These challenges include education, age, income, stigmatisation, and socio-familial support (Pena, Bravo, Tomas, Martínez, Guillen and Jimenez-Ruiz, 2021). They are also highly responsible for and burdened on most or all household activities, including caring for children, husbands, and other family members. There is also an unequal share of responsibilities between women and men, which makes women further disadvantageous in balancing family as well as social life; thus, their socio-economic and health outcomes become severely affected (Mussida and Patimo, 2021). There has been a lack of studies conducted on HIV-positive women’s adherence to HAART in Ethiopia specifically.

Therefore, to address the gap in treatment adherence, this study is designed to develop a better insight into adherence among HIV-positive women on HAART in Southern Ethiopia.

## 2 Methodology

### 2.1 Study design

This study used a descriptive cross-sectional survey in which respondents provided quantitative data via a structured questionnaire to explore and describe factors influencing HAART adherence among HIV-positive women in Southern Ethiopia.

### 2.2 Study area and sampling procedures

The researcher employed a multi-stage sampling method to select the study sites. In the first stage, the Southern Ethiopia Region was randomly selected from the nine regional states of Ethiopia. Following the selection of the region, the researcher compiled a list of all 21 urban public hospitals in the Southern Ethiopia. These hospitals were then stratified into three categories based on their HIV-positive patient load: low, medium, and high volume. The categorization was determined using the median patient volume as the threshold. In the final stage of sampling, one hospital was randomly selected from each of the three strata (low, medium, and high volume), resulting in a total of three urban public hospitals for the study.

### 2.3 Sample size and sampling techniques

Sample size was determined based on a single population proportion formula, n = p (1-p) Z^2^/ d^2^ (Aychiluhm et al., 2021). The following assumptions are considered. The study with the hypothesis of 95% (CI), HAART adherence proportion (P) of 0.746 which was taken from the previous study done in Ethiopia, Eastern Tirgray hospitals (Gebreagziabher and Woldemariam, 2020), and with the marginal error (d) of 0.05. Moreover, a 10% non-response rate was added to the calculated size. Therefore, the final sample size for this study was 320.

### 2.4 Population and sampling methods

For this study, respondents were HIV-positive women both adhering and non-adhering (irrespective of their adherence background) to HAART as they were sampled using a systematic random sampling technique.

The study respondents were selected by a systematic random sampling technique in which the researchers created a sampling frame containing the list of HIV-positive women (who fulfil the inclusion criteria) for the three hospitals. The sampling frame refers to a list, map, or other specification of sampling units in the research population from which the researchers select samples for the specific study (Lohr, 2021). The sampling fraction from each of the study hospitals was determined proportionally to the total number of HIV-positive women on HAART who attended the HAART clinic in each study hospital until the end of March 2023. Then, a systematic random sampling method was applied to select study respondents. Systematic random sampling is a technique where the initial sampling point is selected randomly, and then the respondents are selected at regular intervals (Makwana, Engineer, Dabhi et al., 2023). Based on this, the researchers used this technique to select study respondents from each study hospital, where the sampling interval is the total number of HIV-positive women on HAART divided by the number of study respondents to be included in the study from each study hospital.

As a result, the sampling interval was determined for each of the three study hospitals. The sampling interval for each study hospital was 8. Using systematic random sampling, the first respondent for each study hospital was selected randomly. The subsequent respondents were captured by adding the determined number for the subsequent list of patients in the sample frame. Finally, sampling ended with a sample of 320 respondents for the sampled study hospitals.

### 2.5 Data collection tools, procedures and, quality control

A structured interviewer-administered questionnaire was applied. The questionnaire was developed by making modifications (some questions were adapted) to the AIDS Clinical Trial Group (ACTG) self-reported adherence questionnaire based on the literature review on the research topic, and the theoretical framework using the health belief model (HBM). Permission to use the AACTG-adherence questionnaire was obtained from the copyright holder.

The data collection tool was pre-tested on 16 (5% of the total sample size for this study) respondents using a similar procedure to the actual data collection and with a similar target population and urban public hospital, which was not part of the actual study hospitals in Southern Ethiopia. Moreover, the respondents (HIV-positive women) who participated in the pre-testing were not involved in the actual study. According to the pre-testing results, the data collection instrument (questionnaire) was revised (some questions were adapted) and developed to the current form.

The data collection was done by eight trained and supervised BSc holder nurses using an interviewer-administered questionnaire (data collection tool) at the HAART clinics of the three identified study hospitals from June to July 2023. The researchers conducted the data collectors’ training for 2 days. Important topics including the purpose and objectives of the research, the characteristics of in-person interviews, and interviewing procedures were covered throughout the training and practised via role-playing exercises. The research’s ethical considerations were also covered. The interviewers (the research assistants) and the researchers signed a document committing the interviewers not to record any respondent details on a questionnaire, including names and identification numbers.

The researchers carried out daily supervision to ensure each data collection tool was completed properly.

### 2.6 Data processing and analysis procedure

The data was initially coded, cleaned, and entered into SPSS Version 27 computer software by the researcher. Using descriptive statistics, background factors including sociodemographic and economic data were examined. Bivariable logistic regression analysis was also used to calculate the crude odds ratio (COR) and the adjusted odds ratio (AOR) with 95% CI.

To estimate the occurrence and strength of association, those variables with a *p*-value of < 0.25 were fitted to multivariable logistic regression. In the final model of logistic regression, namely, multivariable analysis, variables were considered significantly associated with HAART medication adherence at a *p*-value less than 0.05.

## 3 Results

### 3.1 Sociodemographic characteristics of respondents

The total sample size of the study was 320. Of these, four respondents discontinued their interview due to the time they would have to spend in the interview, five refused to participate in the study, and five questionnaires were excluded from the analysis because of missing values for key research variables. Therefore, only 306 (N = 306) completed questionnaires were eligible for analysis. The overall response rate was 95.6%, which keeps with the assumption of a 10% non-response rate of the study. The age groups of the respondents were divided into four categories ranging from 18 to 24 years to ≥45 years. The respondents’ demographic data indicates that the mean age was 36.2 years (SD = ±9.1). The minimum age was 19, and the maximum age was 57 years. Regarding the marital status, 150 (49%) respondents were married. About 135 (44.1%) of the respondents were Orthodox Christian in their religion. Hundred seventy-three i.e., 56.5% of the respondents had completed primary/first cycle (grades 1–8) education level. One-third (33%) of the respondents were privately employed and 59 (19.28%) of them were housewives. Regarding the monthly income, the majority, i.e., 148 (48.37%) of them earned 500–2,500 Ethiopian Birr ($50) as a monthly income. Sociodemographic characteristics of respondents are described in detail in Table 1.

**TABLE 1:** Sociodemographic characteristics of HIV-positive women taking HAART at the three urban public hospitals (N = 306).

Variables	Category	Frequency	Percentages
Age in Year	18–24	34	11.1
	25–34	95	31.5
	35–44	116	37.9
	≥45	61	19.3
Marital Status	Married	150	49
	Single/Never married	75	24
	Divorced/Separated	41	13
	Widowed	40	13
Religion	Orthodox	135	44.1
	Protestant	124	40.5
	Muslim	35	11.4
	Catholic	12	3.9
Educational Level	Never attended school	26	8.5
	Primary education	173	56.5
	Secondary education and above	107	35
Occupation	Government’s employee	59	19.28
	Private employee	100	33
	Trader	43	14.05
	Unemployed	45	14.7
	Housewife	59	19.28
Monthly income	500–2,500 Birr ($50)	148	48.37
	2,501–5,000 Birr ($51–100)	111	36.27
	>5,000 Birr ($100)	47	15.36

### 3.2 Level of adherence to HAART medication among women

The study revealed that 253 (82.7%) respondents adhered well to their HAART medication, while 53 (17.33%) respondents had poor adherence to their HAART medication. Therefore, the level of HAART medication adherence in this study was found to be 82.7%.

### 3.3 HAART medication and adherence-related characteristics

In this study, 222 (72.5%;) respondents had travelled ≥10 km and 84 (27.5%) respondents had a history of <10 km travel to the HAART clinic to collect their medication. About 237 (77.45%) HIV-positive women had disclosed their HIV-positive status to others. Only 69 (22.54%) of them had not disclosed their HIV status to others. The study shows that 287 (93.79%) respondents used reminder devices to take their HAART medication at the right time. Most respondents, namely 262 (85.6%), reported that they had spent more than an hour at the HAART clinic to collect their medication regularly. Only 44 (14.38%) respondents were served in less than an hour by healthcare providers during their clinic visit. Of the total 306 respondents, 208 (67.97%) of them were taking a once-a-day ARV pill, which needs to be taken every 24 h, and the remaining 98 (32.03%) of respondents had taken twice-a-day ARV pills every 12 h. Hundred fifty-seven (51.3%) respondents reported depressive symptoms and, 149 (48.7%) respondents had no history of depressive symptoms. According to the study’s findings, as depicted in Table 2, 171 (55.9%) respondents reported perceived stigma, while 135 (44.1%) respondents had no history of perceived stigma. Table 2 indicates HAART medication and adherence-related characteristics of HIV-positive women.

**TABLE 2:** HAART medication and adherence-related characteristics (N = 306).

Variable	Category	Frequency	Percent
Distance (travelled) to the HAART clinic	≥10 km	222	72.5
	<10 km	84	27.5
HIV status disclosure	Yes	237	77.45
	No	69	22.55
Using reminders	Yes	287	93.79
	No	19	6.21
Clinic waiting time	<60 min	44	14.38
	>60 min	262	85.62
Number of times pills taken per day	Once	208	67.97
	Twice	98	32.03
Following a specific HAART medication schedule	Never	16	5.23
	Sometimes	34	11.1
	Half of the time	4	1.31
	Most of the time	184	60.1
	All of the time	68	22.2
Alcohol intake	Yes	70	22.89
	No	236	77.12
Chewing khat	Yes	27	8.8
	No	279	91.2
Depression	Yes	157	51.3
	No	149	48.7
HIV perceived stigma	Yes	171	55.9
	No	135	44.1
Social support from others	Yes	158	51.6
	No	148	48.36
Patient-provider relationship	Good	135	44.1
	Poor	171	55.9

### 3.4 Perception towards HAART adherence among HIV-positive women

In this study, 153 (50%) respondents had a high perceived susceptibility score and the remaining 153 (50%) had a low perceived susceptibility score about HIV disease progression. About 148 (48.36%) respondents had a low perception of the severity of HIV disease and poor HAART medication adherence. About 155 respondents (50.7%) had a low perception of the benefit of good adherence to HAART medication. This study indicated that 167 (54.6%) respondents had a low perception of the barriers to HAART medication adherence. This study indicated that 179 (58.49%) respondents had low self-efficacy about the capability and confidence of taking their HAART medication. This study indicated that 163 (53.26%) respondents had low cues to action. Table 3 indicates the perception towards HAART adherence among HIV-positive women taking HAART.

**TABLE 3:** Perception towards HAART adherence among HIV-positive women taking HAART (N = 306).

Variable	Category	Frequency	Percent
Perceived susceptibility to HIV disease progression	High	153	50
	Low	153	50
Perceived severity of HIV disease and poor adherence	High	158	51.63
	Low	148	48.36
Perceived benefit of adherence to HAART	High	151	49.3
	Low	155	50.7
Perceived barrier	High	139	45.4
	Low	167	54.6
Perceived self-efficacy about the capability and confidence of taking HAART	High	127	41.5
	Low	179	58.49
Cues to action	High	143	46.73
	Low	163	53.26

### 3.5 Reasons for missing any of the HAART medication doses

**FIGURE 1:**
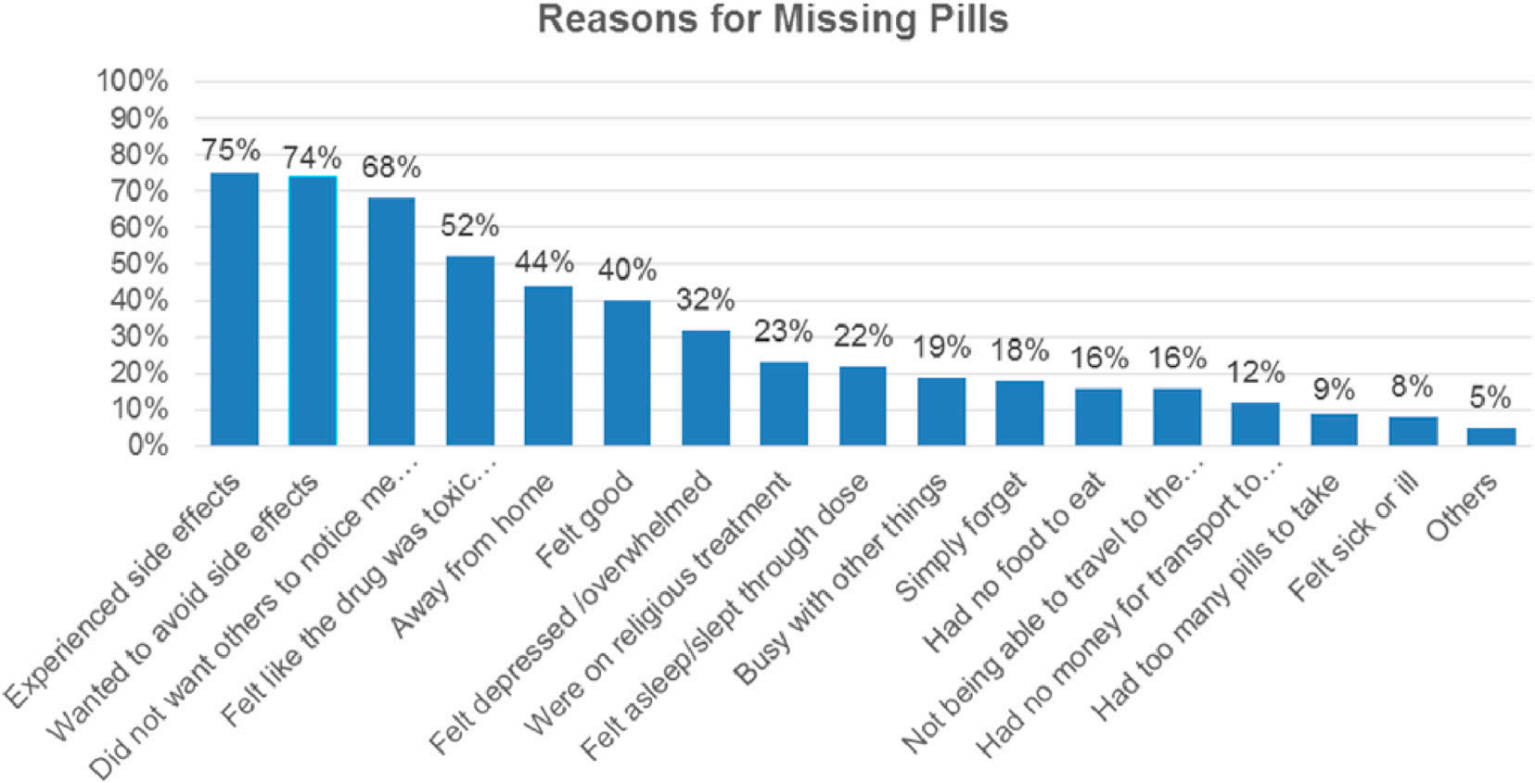
Reasons for missing any of the HAART medication doses repoeted by HIV-positive women (n = 293).

Respondents were asked questions about the reason for missing any dose of HAART medication if they had missed them during the last 30 days. Reasons for missing any of the HAART medication doses reported by HIV-positive women are shown in Figure 1.

Many respondents missed their HAART pills for various reasons. One of the most common reasons reported by the respondents was side effects from HAART medication. 220 (86.92%) respondents were experiencing side effects from the HAART medication. Similarly, 218 (74.4%) respondents reported missing their HAART pills to avoid potential side effects. Additional findings indicated that 199 (67.92%) respondents missed their pills as they did not want others to know about their HIV status.

As the study revealed, 153 (52.2%) HIV-positive women reported that the reason for missing their HAART medication was feeling that the medication was toxic or harmful. 130 (44.36%) respondents reported that being away from home was the reason for missing their pills. 117 (39.93%) respondents reported that they were feeling good and did not think they needed to continue taking the medication as the reason for missing their pills. 95 (32.4%) respondents reported feeling depressed or overwhelmed as the reason for missing their pills. 67 (22.8%) respondents reported undergoing religious treatment as a reason for missing their HAART medication. The least commonly reported reason for not taking or missing their pills was a change in daily routine. The other two reasons that respondents did not mention for missing their pills were traditional medicine and running out of pills.

Other respondents indicated various issues as possible reasons for HIV-positive individuals’ missing HAART pills. These included simply forgetting, not wanting others to know about their HIV status, having too many pills to take, being away from home, feeling sick or ill and being busy. There is a similarity to other studies in Ethiopia (Rike et al., 2021; Gebreagziabher and Woldemariam, 2020; Sharma et al., 2022) in mentioning these excuses. Forgetfulness and being away from home were also mentioned in another study as reasons for missing HAART pills (Zewude and Ajebe, 2022). A personal factor, majorly forgetfulness, was mentioned as the reason for missing pills in the research by Akani (2021).

### 3.6 Factors associated with poor adherence to HAART medication

Several reasons are linked to poor adherence to HAART medication. Numerous investigations have led to various conclusions about the variables influencing adherence to HAART medication. These factors could be socio-demographic, personal-related, medication-related and healthcare service-related. In this study, depression was assessed through the depression assessment questionnaire, which contains seven items. Each item of the depression assessment questionnaire was measured using a 5-point Likert scale, ranging from “never” to “always.” Responses to each questionnaire item were computed as mean score and standard deviation (SD). The depression assessment questionnaire had a good internal consistency in this sample, with Cronbach’s alpha of 0.74. Therefore, the respondents who scored more or equal to the mean score were classified as “respondents with depressive symptoms”, and those who scored less than the mean score were classified as “respondents without depressive symptoms. Inferential statistics to investigate reasons influencing adherence to HAART medication, including bivariable and multivariable logistic regression were conducted.

In the final model of logistic regression, namely, multivariable analysis, six variables were significantly associated with HAART medication adherence at a *p*-value less than 0.05 (Table 4).

**TABLE 4:** Multivariable analysis for factors associated with poor adherence to HAART medication.

Variables	Category	Adherence		Adjusted OR (95%CI)
		Good	Poor	
Marital status	Married	128	22	Ref.
	Never married	65	10	1.09 (0.39–3.00)
	Divorced/ Separated	27	14	2.94 (1.02–8.44)*
	Widowed	33	7	0.68 (0.19–2.37)
Religion	Protestant	119	16	Ref.
	Orthodox	97	27	2.11 (0.94–4.74)
	Muslim	30	5	1.33 (0.34–5.19)
	Catholic	7	5	1.04 (0.18–5.90)
Educational status	Non-formal education	21	5	1.15 (0.14–8.86)
	Primary	127	36	1.36 (0.22–8.23)
	Secondary	75	9	0.94 (0.13–6.42)
	Collage and above	30	3	Ref.
Occupation	Employee	52	7	Ref.
	Private employee	86	14	0.77 (0.22–2.65)
	Marchant	39	4	0.81 (0.17–3.77)
	Unemployed	30	15	3.11 (0.74–12.9)
	Housewife	46	13	1.56 (0.44–5.52)
Using reminders	Yes	242	45	0.25 (0.06–0.97)*
	No	11	8	Ref.
Family and social support	Yes	135	23	0.53 (0.22–1.28)
	No	118	30	Ref.
Depression	Yes	116	41	2.34 (1.01–5.42)*
	No	137	12	Ref.
Perceived stigma	Yes	133	38	2.37 (1.06–5.34)*
	No	120	15	Ref.
Relationship with health worker	Yes	146	25	0.73 (0.29–1.83)
	No	107	28	Ref.
Perceived susceptibility	High	132	21	Ref.
	Low	121	32	4.1 (1.53–11.1)**
Perceived severity	High	139	19	1
	Low	114	34	2.92 (1.14–7.47)*
Perceived self-efficacy	High	111	16	Ref.
	Low	142	37	1.38 (0.56–3.41)
Cues to action	High	124	19	Ref.
	Low	129	34	1.13 (0.48–2.66)

* Significant at a *p*-value < 0.05 level and ** significant at a *p*-value < 0.001.

In this study, we found statistically significant association between marital status, i.e. divorced/separated HIV-positive women [AOR:2.94, 95% CI: (1.02–8.44)], not using reminders [AOR: 0.25, 95% CI: (0.06–0.97)], depression [AOR:2.34, 95% CI: (1.01–5.42)], perceived stigma [AOR:2.37, 95% CI: (1.06–5.34)]., perceived susceptibility [AOR:4.1, 95% CI: (1.53–11.1)], and perceived severity [AOR:2.92, 95% CI: (1.14–7.47)]. Other factors such as educational status, occupation, the frequency of HAART daily doses, family and social support, and patient-healthcare provider relationships were not associated so strongly with poor adherence to HAART medication.

## 4 Discussion

HIV-positive women who were divorced or separated had less adherence to their HAART medication. The divorced/separated HIV-positive women had almost three times more likelihood of being poor adherent to their HAART medication as compared to those who were married [AOR:2.94, 95% CI: (1.02–8.44)]. This could be due to married women having more family support from their spouse and family, which could help them adhere to their HAART medication; women who separated/divorced from their spouse may feel isolated, loneliness, and lack family support, which could make it challenging for them to adhere to their HAART medication. Family members can remind HIV-positive individuals to take their medication at fixed times regularly so that adherence to HAART medication would be increased (Sharma et al., 2022).

The findings from this study are consistent with a study done in Northwest Ethiopia (Tariku et al., 2022), which reported that widowed respondents were 79% less likely to be adherent to their HAART medication when compared with those who were married. The support of male partners to their wives is highly important in HAART medication adherence as they can support women with economic and social issues, facilitating good adherence (Gebreagziabher and Woldemariam, 2020).

Divorced and widowed respondents had two times increased hazard of poor HAART adherence when compared with married respondents (Eze et al., 2023).

Another factor found to be statistically significant with poor adherence to HAART medication in this study was not using reminders. HIV-positive women who used reminders in their medication-taking practice were 75% less likely to be poor adherent to their HAART medication than those who did not use reminders [AOR: 0.25, 95% CI: (0.06–0.97)]. HIV-positive individuals who are taking HAART medication and using a phone alarm indicated that they got remarkable benefits from the phone alarm, especially during oversleeping as the alarm would wake them up to take their HAART medication at the right time (Dzansi et al., 2020). According to the mixed-method study (Villiera et al., 2022), some HIV-positive individuals reported that had challenges which prevented them from taking their HAART medication at the right time as prescribed by healthcare providers. The current study is consistent with a study done in Northern Ethiopia (Gebretsadik, Gebretnsae, Ftwi et al., 2020), where those HIV-positive women who used a clock as a reminder for their medication had a 2.5 times higher HAART medication adherence than those did not use clock reminder. A study conducted in Ethiopia (Ejigu et al., 2020) reported that using reminders among HIV-positive individuals was significantly associated with HAART adherence. In this study, those HIV-positive individuals taking HAART who used a reminder to take their medication had a five times better chance of being adherent than those who did not use a reminder.

In this study, it was determined that depression had a significant relationship with poor adherence to HAART medication. According to the study finding, HIV-positive women who self-reported depression had a 2.34 times greater chance of being poor adherent to their HAART medication than those who did not report depression [AOR:2.34, 95% CI: (1.01–5.42)]. This study is consistent with another study conducted in Ethiopia, which revealed that HIV-positive individuals who developed depression were 2.5 times less likely to be adherent to their HAART medication when compared with individuals without depression (Gebreagziabher and Woldemariam, 2020). In another similar study conducted in India (Chakraborty, Hershow, Qato et al., 2020), the association between depression and low medication adherence was statistically high.

The data revealed that perceived stigma was one of the main factors that affected how well HIV-positive women followed their HAART regimen. There was a statistically significant association between poor adherence to HAART medication and perceived stigma in this study. Those HIV-positive women who reported perceived stigma were 2.37 times more poorly adherent to their HAART medication than those who did not report perceived stigma [AOR:2.37, 95% CI: (1.06–5.34)]. A cross-sectional study conducted in North-Eastern Ethiopia also discovered evidence that those HIV-positive individuals who did not disclose their HIV status to their families due to the fear of stigma were 88% less likely to adhere to their medication as compared to those who disclosed their status (Legesse and Reta, 2019). The finding of the current study is congruent with a study done in Cambodia (Tuot et al., 2023), which reported that perceived stigma affected the medication-taking practice of HIV-positive individuals and correlated with poor HAART adherence.

According to the study findings, perceived susceptibility was one of the factors that had a significant association with poor adherence to HAART medication. HIV-positive women who self-reported low perceived susceptibility were four times more likely to be poor adherent to their HAART medication than those who self-reported high perceived susceptibility [AOR:4.1, 95% CI: (1.53–11.1)]. The finding of the current study is consistent with a study conducted among HIV-positive women in Singapore, which revealed that the perceived susceptibility was statistically associated with medication adherence (Hosseini et al., 2022). Another similar study which used the health belief model (HBM) to determine factors affecting HAART adherence reported that two constructs of HBM, including perceived susceptibility and perceived severity, were significantly associated with HAART medication adherence (Ashraf and Virk, 2021).

According to the study findings, perceived severity was one of the factors that had a significant association with poor adherence to HAART medication. Similar to the perceived susceptibility, HIV-positive women who self-reported low perceived severity were almost three times more likely to be poorly adherent to HAART medication than those who self-reported high perceived severity [AOR:2.92, 95% CI: (1.14–7.47)]. The finding of the current study is consistent with a study conducted among HIV-positive women in Singapore, which revealed that the perceived severity was statistically associated with medication adherence (Ashraf and Virk, 2021). A similar study conducted to assess medication adherence using HBM revealed that the perceived severity was statistically associated with medication adherence (Ling, Zain, Naffi et al., 2023).

## 5 Conclusion

Poor adherence to HAART medication has multiple negative effects on HIV-positive individual’s health conditions. It reduces the immunological protection of HIV-positive individuals, which, in turn, causes high viral load and drug resistance of the virus. This may cause increased HIV-related hospitalization and higher morbidity and mortality. In order to manage HIV infection effectively, adherence to HAART medication is a critical issue. In line with this, understanding the predictors which affect adherence can help to develop tailored interventions to increase adherence and improve the health outcomes of HIV-positive women.

## Data Availability

The raw data supporting the conclusions of this article will be made available by the authors, without undue reservation.
